# Common mental illness among epilepsy patients in Bahir Dar city, Ethiopia: A cross-sectional study

**DOI:** 10.1371/journal.pone.0227854

**Published:** 2020-01-23

**Authors:** Minale Tareke, Minychil Birehanu, Desalegne Amare, Andargie Abate

**Affiliations:** 1 Psychiatry Department, College of Medicine and Health Science, Bahir Dar University, Bahir Dar, Ethiopia; 2 Nursing Department, College of Medicine and Health Science, Bahir Dar University, Bahir Dar, Ethiopia; Uniformed Services University of the Health Sciences, UNITED STATES

## Abstract

**Background:**

Common mental illness has a substantial impact on seizure control and negatively affects the overall quality of life among individuals with epilepsy. However, there is a dearth of studies that examined the associated factors of common mental illness among epilepsy patients in Ethiopia, particularly in the study area. This study aimed to assess the magnitude and factors associated with common mental disorders in epilepsy patients who attended government health institutions in Bahir Dar city, Ethiopia.

**Method:**

Health institution based cross-sectional study was conducted using a systematic sampling technique among people living with epilepsy in Bahir Dar City Administration. Common mental illness was assessed using a self-reporting questionnaire and a semi-structured questionnaire was employed to collect data on socio-demographic and clinical related characteristics. Data were analyzed using descriptive statistics, univariate logistic regression, and multivariable logistic regression.

**Results:**

The magnitude of comorbid common mental illness among people living with epilepsy was found 35.4%. High magnitude of common mental illness was reported among females (39.9%) when compared to males (32.3%). The most prevalent common mental disorders symptoms include being worried, unhappy feeling, trouble thinking clearly, and difficult to enjoy daily activities. Family history of epilepsy, frequent seizures attacks, side effects of antiepileptic drugs, lack of social support and not adherent to antiepileptic drugs were factors associated with common mental illness.

**Conclusions:**

Common mental illness was found to be prevalent among people living with epilepsy. Therefore, it is recommended that great attention should be given to mental illness besides controlling seizure attacks.

## Introduction

Epilepsy is a neurological disorder manifested by episodic disruption of brain electrical activity associated with abnormal body movement, loss of consciousness, and sensory disturbances. World Health Organization (WHO) estimates that fifty million people around the globe are affected by epilepsy, with more than 80% living in the developing world where human and technological resources for care are extremely limited[1, 2]. It is more common in rural areas, perhaps because of higher rates of untreated epilepsy in children, infectious diseases, and low prenatal and postnatal care[3]. Although, epilepsy is more prevalent among young people and older age greater than 60 years, it is not limited to any age group, sex, geographical area, social status, or race [2].

Mental health is the most important component of health that connected closely with the physical and physiological dynamics of the human body[4]. American Psychiatric Association's Diagnostic and Statistical Manual of Mental Disorders fifth edition (DSM-5) defines “Mental Disorder as a syndrome characterized by a clinically significant disturbance in an individual’s cognition, emotion regulation, or behavior that reflects a dysfunction in the psychological, biological, or developmental processes underlying mental functioning”. Mental disorder is commonly associated with substantial distress or impairment in social, occupational, or other essential activities[5].

Mental illness includes common conditions such as depressive disorder, anxiety disorder, psychotic and bipolar disorder. From the total burden of diseases, the disability caused by mental disorder accounts for 25.3% and 33.5% of all years lived in low and middle-income countries, respectively[6].

Psychiatric illnesses presenting in general health care settings among epilepsy patients can be common mental illness like depression, anxiety, somatoform disorders and severe mental illnesses which refers to a group of mental illnesses causing marked disruption in socio-occupational life i.e. schizophrenia and bipolar disorder. Psychiatric comorbidities occur in about one-third of people with epilepsy during the lifespan, and the incidence of these comorbidities are much greater in high-risk groups such as treatment-resistant seizures[7, 8].

The most prevalent psychiatric diagnoses in epilepsy include mood disorders, anxiety disorders, and psychotic disorders. Depression is the top mental illness among epilepsy patients with lifetime prevalence ranges from 20 to 60%, which is consistently higher than the general population with lifetime prevalence rates of 16–20%. It will be the most prevalent cause of disease burden in the world by the year 2030 according to WHO prediction. Anxiety disorders are thought to be the second most common psychiatric disorder in epilepsy with prevalence rates ranging from 11 to 40%. Depression is known to affect the negatively different domains of life such as to disrupt family and friend’s relationships, low productivity at work, and decrease school performance[6, 9, 10].

Common mental illness, in general, has a substantial impact and negatively affect overall psychosocial functioning, quality of life, employment, seizure control, use of the health care system and contribute to adverse effects of antiepileptic medication and other psychosocial problems among individuals with epilepsy. Despite the high prevalence of common mental illness among epilepsy, they remain under-recognized and improperly treated [11].

On the other hand, effective recognition and treatment of common mental illness can improve seizure control among people living with epilepsy. However, the prevalence and factors that might increase common mental illness in patients with epilepsy are not determined well in developing countries, including in Ethiopia. This study aimed to assess the comorbidity of common mental illness among epilepsy patients and the association of this illness with seizure-related and socio-demographic variables.

## Methods

### Study design and period

The institution-based cross-sectional study was done from May-April, 2016 in Bahir Dar city administration, Northwest Ethiopia.

### Study area and population

The study was conducted in Bahir Dar city administration which is the capital city of Amhara regional state. Bahir Dar city is located in Northwest Ethiopia around 565 kilometers from Addis Ababa, the capital city of Ethiopia. The city has a total of 180,174 populations; of these 93,014 are females. The city is divided into 9 sub cities and 17 kebeles. Currently, there are four hospitals (two public and two private), ten health centers (HCs) and many other private health institutions (clinics, pharmacies, and drug shops). The study was carried out in all public health institutions found in the city. The study population was epilepsy patients (≥ 18 years old) who attended the outpatient department during the study period.

### Inclusion and exclusion criteria

All people aged 18 years and above who have treatment follow up and volunteer for the interview were included during the study period. Those who were unable to be interviewed because of intellectual disability or communication difficulties were excluded from participating in the study.

### Sample size determination and procedure

The sample size was determined by using a formula (n = (Z_α/2_)^2^ p (1-p)/ d^2^) for estimating a single population proportion. Considering 95% Confidence Interval (CI) (Z_α/2_ = 1.96), 5% margin of error and due to absence of data in the country, the proportion of population living with epilepsy and who had common mental illness was assumed 50%, generated a minimum sample size of 384 for the study. By adding 10% contingency for non-response, a total of 423 study populations were involved.

A systematic random sampling technique was used to select the study subjects in each health institution. Based on the number of epilepsy patients who attended each health institution, the proportional allocation of the total sample size was carried out to attain the required sample size in each health institution.

### Data collection procedures and quality control

Data was collected using a semi-structured questionnaire which was translated into the local language Amharic and then translated back into English. The questionnaire comprised sociodemographic characteristics, clinical factors, psychosocial and substance use variables. The dose of antiepileptic drugs, taking a single or combination of anti-epileptic drugs, was collected by reviewing the patient’s chart.

Common mental disorders were assessed using Self-Reporting Questionnaire-20(SRQ-20). The SRQ-20 has been developed by world health organization as an instrument to screen mental health problems in primary healthcare attendants in developing countries[12]. It has been previously validated[13] and used in several studies in Ethiopia[14–18]. The SRQ-20 reflects depressive symptoms, anxiety symptoms, and psychosomatic complaints and has been found to detect probable cases of common mental illness with reasonable accuracy. Each of 20 items is scored 0 or 1.A score of 1 indicates that the symptoms were present during the past month, a score of 0 indicates that the symptoms were absent, with a maximum score of 20[12].

Participants who responded eight or more items positively were regarded as having common mental illness as suggested by other studies[18–22]. The internal consistency of the SQR-20 scale was high (Cronbach’s alpha = 0.91) in the current study.

Social support was assessed using the Oslo 3 item social support scale[23]. The sum of social support Oslo 3 item raw scores has a range from 3–14. A score ranging between 3 and 8 is classified as poor support, a score between 9 and 11 as moderate support, and a score between 12 and 14 as strong support. Lastly, we assessed the current use of substances by asking the respondent whether they used or not at least one of the specified substances in the last three months (alcohol, cigarette smoking, chat chewing).

Three clinical nurses and one BSc nurse were responsible to collect data and supervision, respectively for around one-month duration. Two-day training was prepared to orient data collectors and supervisor on the questionnaire to be used, the purpose of the study and how to approach respondents and obtain consent. The questionnaire was pre-tested on 5% randomly selected samples from nearby health center patients. The respondents were interviewed in their language (Amharic). The supervisor was checked for the completeness of questionnaires every day.

### Data handling and analysis

The collected data were entered into computers by using Epi- Info version 7 and transferred to SPSS version 20 software computer program. Percentage, frequency, and mean were used to describe the study participants concerning relevant variables. Logistic regression was performed to assess the association between binary outcomes and different explanatory variables. Bivariate analysis was first conducted for each potentially explanatory risk factor. Variables that satisfied p-value < 0.2 were selected for further analysis using multiple logistic regression models. The strength of association was interpreted using the odds ratio and confidence interval. P < 0.05 was considered statistically significant in this study.

### Ethical consideration

Ethical clearance was obtained from the Research Ethical Review Committee of Bahir Dar University College of Medicine and Health Sciences, and permission and supporting letter from Amhara regional state health bureau. The letter was submitted to each health institution before data collection. Written informed consent from each study participants were obtained after a clear explanation about the purpose of the study during data collection. The respondents were given the right to refuse to take part in the study as well as to withdraw at any time during the study. Refusal to participate did not result in loss of medical care provided or any other benefits. Privacy and confidentiality were maintained throughout the study by interviewing the patient alone and by using code instead of the name.

## Results

### Socio-demographic characteristics

Three hundred ninety-eight (398) people living epilepsy were involved in the study, with the response rate of 94%. The mean (standard deviation) age of the respondents was 28.6 (±10.5) years. Around fifty-nine percent (59.0%) of the respondents were males. Almost half of the respondents were single (50.5%) (Table 1).

**Table 1:** Distribution of participants by socio-demographic characteristics at government health institution in Bahir Dar City, 2017(n = 398).

Variable		Number	Percentage (%)
Sex	Male	235	59.0
	Female	163	41.0
Age	18–24	172	43.2
	25–34	136	34.2
	35+	90	22.6
Marital status	Married	159	39.9
	Single	201	50.5
	Divorced/Widowed	38	9.5
Residence	Urban	220	55.3
	Rural	178	44.7
Educational status	Unable to write and read	134	33.7
	From grade 1–8	129	32.4
	From grade 9–12	84	21.1
	Diploma and above	51	12.8
Occupation	Government employee	30	7.5
	Merchant	29	7.3
	Farmer	94	23.6
	Day labor	53	13.3
	House wives	23	5.8
	Private employee	76	14.6
	No Job	47	11.8
	Others	64	16.1
Monthly income (Ethiopian birr)	<500	94	23.6
	500–799	86	21.6
	800–1408	119	29.9
	>1408	99	24.9
Living condition	Alone	67	16.8
	With family	314	78.9
	With friend/Homeless/others	17	4.3

### Clinical characteristics of the respondents

From a total of 398 respondents, nearly one fifth (18.6%) of the respondents have a family history of epilepsy. Among patients who had a family history of epilepsy, more than half (51.2%) of them had common mental illness comorbidity. From chart review, four antiepileptic drugs were prescribed in outpatient clinics. The majority of the respondents were using a single drug (78.1%). Almost all (89.9%) epilepsy patients have taken phenobarbitone and the least prescribed one is phenytoin (4.0%). Phenobarbitone and carbamazepine are the most combinations (13.3%), following phenobarbitone and sodium valproate (4.8%) in this study.

Nearly, one-fifth (19.3%) of the respondents have frequent seizure attacks (one and more attacks per month) and among them,54.5% had psychiatric comorbidity (Table 2).

**Table 2:** Distribution of people living with epilepsy by their clinical status at government health institutions in Bahir Dar City, 2017(n = 398).

Variable Name		Frequency	Percent
Family history of epilepsy	Yes	74	18.6
	No	324	81.4
Duration of illness	≤5 year	159	39.9
	6-10year	121	30.4
	>10 year	118	29.6
Age at the onset of illness	<10 year	58	14.6
	10–19 year	188	47.2
	20–29 year	99	24.9
Frequency of seizure	≥ 30 years	53	13.3
	Frequent(≥1/month)	77	19.3
	1–3 seizures/year	157	39.5
	Seizure free for 1 year	164	41.2
Type of drugs	one	311	78.1
	≥ two	87	21.9
Duration on anti-epileptic drugs	≤5 years	257	64.6
	6–10 years	105	26.4
	>10 years	36	9.0
Side effects of drugs	Yes	153	38.4
	No	245	61.6
Medication price	Free	154	38.7
	Buying	244	61.3
Comorbid of chronic medical disease	Yes	19	4.8
	No	379	95.2
Adherence to drugs	good	307	77.1
	poor	91	22.9
Current substance use (alcohol, chat, cigarette)	Yes	8	2.0
	No	390	98.0

## Magnitude of comorbid common mental illness among people living with epilepsy

The magnitude of comorbid common mental illness among people living with epilepsy who have follow up in Bahir Dar City administration was found to be 35.4% (95% CI: 30.9, 40. 2%). A high prevalence of common mental illness was found among females (39.9%) when compared to males (32.3%) in the current study. The distribution of SRQ-20 showed a mean score of 5.4 (±5.3) with a range from 0 to 19. The most prevalent symptoms of comorbid common mental illness were tense or worried (47.5%), headache (41.2%), unhappy feeling (35.7%) and trouble thinking clearly (34.4%) while the least common symptoms mentioned by respondents were sleeping badly (12.1%) and hands shake (13.6%) (see Table 3).

**Table 3:** Distribution of SRQ-20 symptoms among people living with epilepsy attending at government health institutions in Bahir Dar City, 2017 (n = 398).

SRQ-20 Items	Yes responses Frequency (%)	No responses Frequency (%)
Do you often have headache?	164(41.2)	234(58.8)
Is your appetite poor?	82(20.6)	316(79.4)
Do you sleep badly?	48(12.1)	350(87.9)
Are you easily frightened?	110(27.6)	288(72.4)
Do your hands shake?	54(13.6)	344(86.4)
Do you feel nervous, tense or worried?	189(47.5)	209(52.5)
Is your digestion poor?	81(20.4)	317(79.6)
Do you have trouble thinking clearly?	137(34.4)	261(65.6)
Do you feel unhappy?	142(35.7)	256(64.3)
Do you cry more than usual?	99(24.9)	299(75.1)
Do you find it difficult to enjoy your daily activities?	127(31.9)	271(68.1)
Do you find it difficult to make decisions?	104(26.1)	294(73.9)
Is your daily work suffering?	93(23.4)	305(76.6)
Are you unable to play a useful part in life?	102(25.6)	296(74.4)
Have you lost interest in things?	85(21.4)	313(78.6)
Do you feel that you are a worthless person?	103(25.9)	295(74.1)
Has the thought of ending your life been on your mind?	105(26.4)	293(73.6)
Do you feel tired all the time?	95(23.9)	303(76.1)
Do you have uncomfortable feelings in your stomach?	113(28.4)	285(71.6)
Are you easily tired?	123(30.9)	275(69.1)
**Common mental disorder cases (cut-off point ≥8)**	**141(35.4)**	**257(64.6)**

### Factors associated with co-morbid common mental illness

In multiple logistic regression analysis, having a family history of epilepsy, frequent seizures attacks, side effects of antiepileptic drugs, lack of social support and not adherent to antiepileptic drugs were significantly associated factors of comorbid common mental illness among respondents (Table 4).

**Table 4:** Bivariate and multiple analysis of variables associated with common mental illness among people living with epilepsy at government health institutions in Bahir Dar City, 2017(n = 398).

Variable	Common mental illness			OR with 95% CI	
		Yes	No	Crude	Adjusted
Age	18–24 year	59	113	1	1
	25–34 year	42	94	0.85(0.52,1.38)	1.00(0.55,1.83)
	35 year & above	40	50	1.53(0.91,2.58)	1.34 (0.69,2.59)
Sex	Male	76	159	1	1
	Female	65	98	1.38(0.91,2.10)	1.60(0.95,2.70)
Living condition	With family	109	205	1	1
	Alone	22	45	0.92(0.52,1.61)	1.84(0.91,3.72)
	With friend/homeless	10	7	2.68(0.99,7.25)	1.58(0.40,6.18)
Family history of epilepsy	Yes	38	36	2.26(1.35,3.78)	2.57(1.35,4.91)*
	No	103	221	1	1
Social support	Poor	87	73	7.69(3.79,15.60)	2.94(1.29,6.65)*
	Medium	43	113	2.45(1.18,5.07)	9.05(4.03,20.31)*
	Strong	11	71	1	1
Frequency of seizures	≥ one seizure/month	42	35	4.58(2.55,8.24)	4.53(2.25,9.11)*
	1–3 seizure/year	65	92	2.70(1.64,4.42)	2.47(1.37,4.43)
	No seizure/year	34	130	1	1
Side effect of medication	Yes	74	79	2.48(1.62,3.80)	2.12(1.26,3.55)*
	No	67	178	1	1
Co- morbid disease	Yes	12	7	3.32(1.27,8.64)	2.10(0.65,6.78)
	No	129	250	1	1
Types of drug	One drug	104	207	1	1
	≥two drugs	37	50	0.67(0.41,1.10)	0.99(0.52,1.87)
Adherence to drug	good	77	230	1	1
	Poor	64	27	7.08(4.21,11.89)	6.99(3.83,12.78) *

* = P<0.05

## Discussion

The magnitude of common mental illness among people living with epilepsy who have treatment follow up at government health institutions in Bahir Dar City administration was found to be 35.4% (using SRQ-20 cut-off point of ≥8). Multiple analysis revealed that having the family history of epilepsy, frequent seizures attacks, side effects of antiepileptic drugs, lack of social support and not adherent to antiepileptic drugs were factors associated with common mental illness.

The magnitude of common mental disorders in the current study is in line with the findings done in Mexico[24] (36.4%) and in India (32.5%)[25, 26]. However, this study has revealed much lower magnitude of comorbidity of common mental illness than the result done in Zambia (53.7%)[27], Brazil (54.1%)[28], India (50.0%)[29], and Turkey(40.5%)[30]. The higher prevalence in Zambia and Brazil might be because of differences in inclusion criteria and instrument variation for screening mental illness. For instance, in Brazil, patients with temporal lobe epilepsy were the study population but our study population includes all types of epilepsy. More psychiatric disturbances are found in patients with temporal lobe epilepsy[31], which suggests limbic dysfunction rather than a psychological adjustment to a chronic epileptic condition. The limbic system is located in the medial parts of the temporal lobes and involved in the regulation of emotional behavior[32]. The majority of our study population were from referral hospital which might have better access to detect their mental illness symptoms and managed early by psychiatry professional specialist. However, Zambia’s study was conducted at primary health clinics.

But, this study was somewhat higher than studies done in Australia(24%)[33], Norway(26%)[34] and Sierra Leone(27.5%)[35].People living with epilepsy in high-income countries might be early identified and treated comorbid mental illness simultaneously while controlling seizure attacks. The other difference might be inclusion criteria, study design, and sample size difference. Our study also showed that comorbidity of mental illness found to be greater than the study done in the general population in Ethiopia(27.9%)[36]. This is supported by another study that psychiatric comorbidities are common in epilepsy often comorbid at rates two-three-fold or higher than in the general population without epilepsy[37]. The other reason may be due to comorbid mental illness is often underdiagnosed and undertreated among epilepsy patients[38]. At last, the quality of life is likely to be worse in epilepsy patients with common mental disorders[39].

The present study showed that a family history of epilepsy was associated with mental illness which is consistent with other studies [40, 41]. The commonest (27%) risk factors identified in the hospital-based retrospective study in Saudi Arabia was the presence of a family history of epilepsy[42]. This might contribute to the individuals' perception of illness as they gained from their family. The other reason might be due to the individuals who have a family history of epilepsy may witness seizure attacks that increase mental distress as thinking shortly developing such type of attacks. Moreover, epilepsy and mental illness may share a common genetic predisposition. This is supported by the magnitude of mood disorders that were found high among people living with epilepsy who had a relative with focal epilepsy[41]. Common mental disorders found to be higher in epilepsy patients and people with common mental disorders have been more likely to suffer from epilepsy[39].

This study also showed that poor social support has a significant association to increase odds of developing a mental illness which is supported by another study[43]. Participants who have poor social support was contributed to greater perceived stress and depression. They also go through stressful conditions and unable to cope with this easily[44].Studies revealed that providing good social support can protect individuals from the feeling of suicidal thought and attempt, worthiness during the times of an unstable situation and decreases feelings of despair[45, 46]. This is evidenced in the previous community-based study in Ethiopia showed that providing a high level of psychological support protect the individuals from developing common mental illness by 50%[47].

Frequent seizure attacks were associated with common mental illness which is consistent with other studies[48–51]. Many epilepsy patients become fearful when they have seizure attacks in public place: one of the most awful things for epilepsy patients, felt ashamed about their unpredictable seizure attacks may be associated with socially unaccepted sign like incontinence of feces, urine, jerking movements, bite of tongue or cheek may occur and bloody saliva may come from the mouth. This might be the community attitude towards epilepsy patients which supported the study done in Southwest Ethiopia. The majority (85.3%) of the community mentioned that epilepsy is a mental disease and around half of them believe that the cause of epilepsy was contagious, and cursed from God[52]. This, in turn, leads to having a high level of perceived stigma (71.6%) among epilepsy patients with strong predictors for subsequent frequent seizures[53]. Comorbidities of mental illness were found in more than two-thirds of the patients who have frequent seizure attacks. It was also more common among generalized seizures in contrast to partial seizures[54] and associated with a poor course of the seizure disorder[55]. Frequent seizure attacks increase suicidal ideation and attempt among people living with epilepsy [46]. Comorbid mental illness affects negatively health-related quality of life and clinical course of epilepsy[56].

Poor adherence to epileptic drugs was a strong predictor of common mental illness which is support by other studies[50, 57, 58]. The possible reason might be epilepsy patients with poor adherence to their medication may result in uncontrolled seizures attacks that may contribute patients to have a common mental illness. This is confirmed that frequent seizure attacks are associated with a higher magnitude of mental illness when compared to controlled seizure patients [59]. Epilepsy patients may not take their prescribed drugs due to already existing mental illness. This is supported by patients who have a history of mental illness before treatment was found to stop their new antiepileptic drugs due to mental illness consequences when compared to those who have no previous mental illness diagnosis[60].

In general, the implication of the current research result shows that pro-active mental illness screening, detection, and early treatment should be incorporated in neurologic clinics to control frequent seizure attacks and to have good adherence to their medication. Multidisciplinary efforts are required within different stakeholders to bring overall management of these complex comorbidities. The study generated baseline evidence in Ethiopian context about the burden of comorbid mental illness in epilepsy patients. This is believed to be one of the essential information to provide appropriate treatment and avoid unwanted incorrect perceptions attached to the illness.

Despite the above strength, our limitations include some of the variables reported by the respondents such as a family history of epilepsy, duration of illness and age at the onset of illness may face recall bias. We have difficulty to know the magnitude of mental illness based on seizure type since a specific diagnosis of epilepsy was not written while reviewing the patient chart. In addition, there was difficulty to identify how many people in our study were screened positive for common mental illness and have been received mental health treatment before the time of the data collection period. Finally, we have faced difficulties to identify which occurred first from mental illness and epilepsy because the nature of the study design we used was cross-sectional.

## Conclusion

Compared to the general population, epilepsy patients have a high burden of comorbid mental illness. Family history of epilepsy, frequent seizures attacks, side effects of antiepileptic drugs, lack of social support and not adherent to antiepileptic drugs were factors associated with common mental illness. Common mental illness was found to be prevalent among people living with epilepsy. Therefore, it is recommended that great attention should be given to comorbid mental illness in neurology clinics to control frequent seizure attacks and to make the patient more adhere to their treatment. It is also recommended follow up study to see whether the burden of mental illness before or after the diagnosis of epilepsy concerning the impact of mental illness in neurologic clinics on the overall quality of life.

## Supporting information

S1 Dataset (ZIP)

## References

[1] WHO. Epilepsy in the WHO eastern Mediterranean region: Bridging the gap. 2010.

[2] Mula M. Neuropsychiatric Symptoms of Epilepsy. Switzerland Springer International Publishing; 2016. 387 p.

[3] MF BEAR, CONNORS BW, PARADISO MA. NEUROSCIENCE: EXPLORING THE BRAIN. FOURTH EDITION ed: Wolters Kluwer; 2016 650–60 p.

[4] Tawar S, Bhatia S, Ilankumaran M. Mental health, are we at risk?. Indian J Community Med 2014;39(1):43–6. 10.4103/0970-0218.126359 24695680 PMC3968582

[5] Association AP. Diagnostic and Statistical Manual of Mental Disorders. Fifth Edition ed. Washington, D.C: American Psychiatric Association; 2013.

[6] WHO. Global burden of mental disorders and the need for a comprehensive, coordinated response from health and social sectors at the country level. 2011:1–6.

[7] Keezer MR, Sisodiya SM, Sander JW. Co morbidities of epilepsy: current concepts and future perspectives. Lancet Neurol. 2015:1–10. 10.1016/S1474-4422(15)00225-2 26549780

[8] Mula M. Treatment issues for psychiatric comorbidities of epilepsy. Clin Pract. 2013;10(3):293–9.

[9] Sezibera V, Nyirasafari D. Incidence of depression in Epilepsy patients. Rwanda Journal, Series F: Health Sciences. 2013;1(1):67–77.

[10] Barr WB, Morrison C. Clinical Handbooks in Neuropsychology of Epilepsy. New York Springer Science+Business Media; 2015 224–35 p.

[11] Fiest KM, Patten SB, Jetté N. Screening for Depression and Anxiety in Epilepsy. Neurol Clin 2015:1–11. 10.1016/j.ncl.2014.09.010 27086983

[12] WHO. A user's guide to the self Reporting Questionnaire (SRQ). Geneva, Switzerland. 1994.

[13] Hanlon C, Medhin G, Alem A, Araya M, Abdulahi A, Hughes M, et al Detecting perinatal common mental disorders in Ethiopia: Validation of the self-reporting questionnaire and Edinburgh Postnatal Depression Scale. Journal of Affective Disorders. 2008;108:251–62. 10.1016/j.jad.2007.10.023 18055019

[14] MOGGA S, PRINCE M, ALEM A, KEBEDE D, STEWART R, GLOZIER N, et al Outcome of major depression in Ethiopia. B R I T IS H J O UR N A L O F P SYC HIAT RY 2006;189:2 4 1–2 4 6 10.1192/bjp.bp.105.013417 16946359

[15] Hanlon C, Medhin G, Alem A, Araya M, Abdulahi A, Tomlinson M, et al Sociocultural practices in Ethiopia: association with onset and persistence of postnatal common mental disorders. The British Journal of Psychiatry 2010;197:468–75. 10.1192/bjp.bp.109.076497 21119153 PMC2994937

[16] Gelaye B, Lemma S, Deyassa N, Bahretibeb Y, Tesfaye M, Berhane Y, et al Prevalence and Correlates of Mental Distress Among Working Adults in Ethiopia. Clinical Practice & Epidemiology in Mental Health. 2012;8:126–33. 23166564 10.2174/1745017901208010126 PMC3496909

[17] Dessie Y, Ebrahim J, Awoke T. Mental distress among university students in Ethiopia: a cross sectional survey. Pan African Medical Journal. 2013;15(95). 10.11604/pamj.2013.15.95.2173 PMC3810159 24198889

[18] Zeleke W, Minaye A, Kanyongo GY. Mental Health and Somatic Distress among Ethiopian Migrant Returnees from the Middle East. Int J Ment Health Psychiatry 2015;1(2):1–6.

[19] ALEM A. Prevalence of mental distress in the outpatient clinic of a specialized leprosy hospital. Addis Ababa, Ethiopia, 2002. Lepr Rev. 2004;75:367–75. 15682974

[20] Selamawit Z, Nurilign A. Common mental disorder among HIV infected individuals at Comprehensive HIV Care and Treatment Clinic of Debre Markos referral Hospital, Ethiopia. Journal of AIDS and Clinical Research. 2015;6(2).

[21] Melese B, Bayu B, Wondwossen F, Tilahun K, Lema S, Ayehu M, et al Prevalence of mental distress and associated factors among Hawassa University medical students, Southern Ethiopia: a cross-sectional study. BMC research notes. 2016;9(1):485 10.1186/s13104-016-2289-7 27821143 PMC5100187

[22] Getinet Alemu W, Dessalegn Malefiya Y, Boru Bifftu B. Mental Distress among Patients Admitted in Gondar University Hospital: A Cross Sectional Institution Based Study. Health Science Journal. 2016;10(6).

[23] Baskind R, Birbeck GL. Epilepsy-associated stigma in sub-Saharan Africa:The social landscape of a disease. Epilepsy Behav 2005;7:68–73. 10.1016/j.yebeh.2005.04.009 15978874

[24] Domínguez-Aguilera MC, Muniz-Landeros ˜ CE. Prevalence of psychiatric disorders in patients with epilepsy in a tertiary level care hospital: Detection through the MINI PLUS International Structured Interview. Medicina Universitaria 2017.

[25] Amruth G, Praveen-kumar S, Nataraju B, Kasturi P. Study of psychiatric comorbidities in epilepsy by using the Mini International Neuropsychiatric Interview. Epilepsy & Behavior 2014;33:94–100. 24632481 10.1016/j.yebeh.2014.02.001

[26] Alsaadi T, El Hammasi K, Shahrour TM, Shakra M, Turkawi L, Almaskari B, et al Prevalence of depression and anxiety among patients with epilepsy attending the epilepsy clinic at Sheikh Khalifa Medical City, UAE: A cross-sectional study. Epilepsy & Behavior. 2015;52:194–9. 26448591 10.1016/j.yebeh.2015.09.008

[27] Mbewe EK, Uys LR, Nkwanyana NM, Birbeck GL. A primary healthcare screening tool to identify depression and anxiety disorders among people with epilepsy in Zambia. Epilepsy & Behavior. 2013;27(2):296–300. 23510742 10.1016/j.yebeh.2013.01.025 PMC3628275

[28] Bragatti JA, Torres CM, Londero RG, Assmann JB, Fontana V, Martin KC, et al Prevalence of psychiatric comorbidities in temporal lobe epilepsy: the value of structured psychiatric interviews. Epileptic disorders. 2010;12(4):283–91. 10.1684/epd.2010.0345 21112827

[29] Rani R, Arora R, Dass Garg P, Bala N, Neki NS. Prevalence of psychiatric comorbidities among the patients of epilepsy attending general hospital psychiatric unit. Int J Curr Res Med Sci 2018;4(5):90–6.

[30] Altınöz AE, Meriç OT, Altınöz ŞT, Eşsizoğlu A, Coşar B. Psychiatric disorders comorbid with epilepsy in a prison sample. Seizure. 2016;40:133–5. 10.1016/j.seizure.2016.06.016 27423133

[31] Henning O, Nakken K. Psychiatric comorbidity and use of psychotropic drugs in epilepsy patients. Acta Neurologica Scandinavica. 2010;122:18–22. 10.1111/j.1600-0404.2010.01370.x 20586730

[32] Swinkels WA, Kuyk JV, Van Dyck R, Spinhoven PH. Psychiatric comorbidity in epilepsy. Epilepsy & Behavior. 2005;7(1):37–50. 15975853 10.1016/j.yebeh.2005.04.012

[33] Lacey CJ, Salzberg MR, Roberts H, Trauer T, D’Souza WJ. Psychiatric comorbidity and impact on health service utilization in a community sample of patients with epilepsy. Epilepsia. 2009;50(8):1991–4. 10.1111/j.1528-1167.2009.02165.x 19490049

[34] Henning O, Nakken K. Psychiatric comorbidity and use of psychotropic drugs in epilepsy patients. Acta Neurologica Scandinavica. 2010;122(s190):18–22. 10.1111/j.1600-0404.2010.01370.x 20586730

[35] M’bayo T, Tomek M, Kamara C, Radcliffe Lisk D. Psychiatric comorbidity in African patients with epilepsy–Experience from Sierra Leone. Int J Epilepsy. 2017;61:1–5. 10.1016/j.ijep.2016.12.002

[36] Fekadu A, Medhin G, Selamu M, Hailemariam M, Alem A, Giorgis TW, et al Population level mental distress in rural Ethiopia. BMC psychiatry. 2014;14(1):194. 24999041 10.1186/1471-244X-14-194 PMC4227077

[37] Josephson CB, Jetté N. Psychiatric comorbidities in epilepsy. International Review of Psychiatry. 2017. 10.1080/09540261.2017.1302412 28681667

[38] Verrotti A, Carrozzino D, Milioni M, Minna M, Fulcheri M. Epilepsy and its main psychiatric comorbidities in adults and children. Journal of the neurological sciences. 2014;343(1–2):23–9. 10.1016/j.jns.2014.05.043 24929650

[39] Kwon OY, Park SP. Depression and anxiety in people with epilepsy. Journal of clinical neurology. Journal of clinical neurology. 2014;10(3):175–88. 10.3988/jcn.2014.10.3.175 25045369 PMC4101093

[40] Hesdorffer DC, Caplan R, Berg AT. Familial clustering of epilepsy and behavioral disorders: evidence for a shared genetic basis. Epilepsia. 2012;53(2):301–7. 10.1111/j.1528-1167.2011.03351.x 22191626 PMC3267857

[41] Insel BJ, Ottman R, Heiman GA. Mood disorders in familial epilepsy: A test of shared etiology. Epilepsia. 2018;59(2):431–9. 10.1111/epi.13985 29318616 PMC5803440

[42] Babtain FA. Impact of a family history of epilepsy on the diagnosis of epilepsy in southern Saudi Arabia. Seizure 2013;22:542–7. 10.1016/j.seizure.2013.04.002 23628167

[43] Wang J, Mann F, Lloyd-Evans B, Ma R, Johnson S. Associations between loneliness and perceived social support and outcomes of mental health problems: a systematic review. BMC psychiatry. 2018;18(1):156 10.1186/s12888-018-1736-5 29843662 PMC5975705

[44] Mama SK, Li Y, Basen-Engquist K, Lee RE, Thompson D, Wetter DW, et al Psychosocial mechanisms linking the social environment to mental health in African Americans. PloS one. 2016;11(4):e0154035 10.1371/journal.pone.0154035 27119366 PMC4847864

[45] Letvak S. The importance of social support for rural mental health. Issues in mental health nursing. 2002;23(3):249–61. 10.1080/016128402753542992 11942190

[46] Haile K, Awoke T, Ayano G, Tareke M, Abate A, Nega M. Suicide ideation and attempts among people with epilepsy in Addis Ababa, Ethiopia. Annals of general psychiatry. 2018;17(1):4. 29410698 10.1186/s12991-018-0174-6 PMC5782394

[47] Yimam K, Kebede Y, Azale T. Prevalence of common mental disorders and associated factors among adults in Kombolcha Town, Northeast Ethiopia. Journal of Depression and Anxiety S. 2014;1:2167–1044.

[48] Tsegabrhan H, Negash A, Tesfay K, Abera M. Co-morbidity of depression and epilepsy in Jimma University specialized hospital, Southwest Ethiopia. Neurology India. 2014;62(6):649 10.4103/0028-3886.149391 25591679

[49] Tegegne MT, Mossie TB, Awoke AA, Assaye AM, Gebrie BT, Eshetu DA. Depression and anxiety disorder among epileptic people at Amanuel Specialized Mental Hospital, Addis Ababa, Ethiopia. BMC Psychiatry 2015;15(210). 10.1186/s12888-015-0589-4 26328614 PMC4556015

[50] Bifftu BB, Dachew BA, Tiruneh BT, Tebeje NB. Depression among people with epilepsy in Northwest Ethiopia: a cross-sectional institution based study. BMC research notes. 2015;8(1):585. 26482788 10.1186/s13104-015-1515-z PMC4617742

[51] Chandrasekharan SC, Menon V, Wadwekar V, Nair PP. High frequency of depressive symptoms among adults with epilepsy: Results from a Hospital-based study. Journal of neurosciences in rural practice. 2017;8(Suppl 1):S13 10.4103/jnrp.jnrp_21_17 28936065 PMC5602239

[52] Henok A, Lamaro T. Knowledge about and attitude towards epilepsy among Menit Community, Southwest Ethiopia. Ethiopian journal of health sciences. 2017;27(1):47–58. 10.4314/ejhs.v27i1.7 28458490 PMC5390228

[53] Bifftu BB, Dachew BA, Tiruneh BT. Perceived stigma and associated factors among people with epilepsy at Gondar University Hospital, Northwest Ethiopia: a cross-sectional institution based study. African health sciences. 2015;15(4):1211–9. 10.4314/ahs.v15i4.21 26958023 PMC4765415

[54] Charfi N, Daoud S, Farhat N, Bouali MM, Zouari L, Zouari N, et al Prevalence of psychiatric comorbidities in epilepsy. European Psychiatry. 2017;41:S470.

[55] Kanner AM. Management of psychiatric and neurological comorbidities in epilepsy. Nature Reviews Neurology. 2016;12(2):106 10.1038/nrneurol.2015.243 26782334

[56] Li T. Epilepsy and associated comorbidities. Neuropsychiatry (London). 2017;1(1):1–3.

[57] O’Rourke G, O’Brien JJ. Identifying the barriers to antiepileptic drug adherence among adults with epilepsy. Seizure. 2017;45:160–8. 10.1016/j.seizure.2016.12.006 28063375

[58] Chaka A, Awoke T, Yohannis Z, Ayano G, Tareke M, Abate A, et al Determinants of depression among people with epilepsy in Central Ethiopia. Annals of general psychiatry. 2018;17(1):27. 29942342 10.1186/s12991-018-0197-z PMC5998556

[59] Jackson M, Turkington D. Depression and anxiety in epilepsy. Journal of Neurology, Neurosurgery & Psychiatry. 2005;76(suppl 1):i45–i7. 10.1136/jnnp.2004.060467 PMC1765680 15718221

[60] Stephen LJ, Wishart A, Brodie MJ. Psychiatric side effects and antiepileptic drugs: observations from prospective audits. Epilepsy & Behavior. 2017;71:73–8. 28551500 10.1016/j.yebeh.2017.04.003

